# Is diabetes mellitus a risk factor for low bone density: a systematic review and meta-analysis

**DOI:** 10.1186/s12902-021-00728-3

**Published:** 2021-04-13

**Authors:** Jingying Qiu, Chengjiang Li, Zhichun Dong, Jing Wang

**Affiliations:** 1 Department of Endocrinology, Shengzhou People’s Hospital (The First Affiliated Hospital of Zhejiang University Shengzhou Branch, Zhejiang, China), No. 666, Dangui Road, Shengzhou, 312400 Zhejiang China; 2 Department of Endocrinology, The First Affiliated Hospital Zhejiang University, Hangzhou, Zhejiang China

**Keywords:** Osteoporosis, Diabetes mellitus, Systematic review, Meta-analysis, Risk factors

## Abstract

**Background:**

This systematic review aimed to investigate whether diabetes mellitus is a risk factor for low bone density, as this might be important and necessary for doctors specialized in treating patients with low bone density.

**Methods:**

PubMed, Embase, CINAHL, and SciELO were searched for cohort, case-control, and cross-sectional studies that investigated the effects of diabetes mellitus on bone mineral density till January 2020. Data screening and extraction are done independently, whereas the methodological quality of the studies was assessed according to the Newcastle-Ottawa Scale (NOS).

**Results:**

A total of 14 studies that met the eligibility criteria including 24,340 participants were enrolled. The overall quality of the studies had a scale of over 6 points. The overall odds ratio (OR) regarding the risk of diabetes mellitus in low bone density patients was 1.20 [95% confidence interval (CI)0.80–1.79, *P* = 0.30], and type 2 diabetes mellitus (T2DM) (OR = 0.69 [0.11, 4.55], *P* = 0.70). Subgroup analysis revealed that whether females or males, developed or developing countries, T2DM, studies after 2015, and quality over 7 points (all *P* values > 0.05) showed no significant differences with the risk of low bone density, except type 1 diabetes mellitus (T1DM) (OR = 3.83 [1.64, 8.96], *P* = 0.002), and studies before 2015 (OR = 1.76 [1.06, 2.92], *P* = 0.03), and quality below 7 points (OR = 2.27 [1.50, 3.43], *P* = 0.0001). Funnel plot showed no significant asymmetry.

**Conclusions:**

These findings revealed no relationship between T2DM and low bone density, and also, the evidence between T1DM and low bone density is inadequate, requiring further analysis of well-designed cohort studies.

## Background

Osteoporosis is a chronic metabolic bone disease that is characterized by decreased bone mass and deterioration of microarchitectural bone tissue, which leads to fractures in individuals [1]. It has been reported that 50% of women and 20% of men over 50 years of age experience osteoporotic-related fracture, causing morbidity and mortality [2]. The imbalance in the activities of osteoclasts and osteoblasts resulted in decreased bone mineral density and irreversible bone mass loss by accelerating bone resorption and/or slow bone formation [3, 4]. Clinically, low bone density most often occurs through estrogen reduction in postmenopausal women and age-related bone loss in females [5, 6]. Other risk factors of osteoporosis included gene, cigarette smoking, alcohol consumption, abnormally high plasma serum parathyroid hormone (PTH) levels, physical inactivity, and chronic use of some medications, for example, corticosteroids [7–9]. Inadequate physical activity always leads to a sedentary lifestyle in the elderly, paralyzed, or limited activity due to accelerated bone loss [10–12].

Diabetes mellitus is a chronic metabolic disease, especially type 2 diabetes mellitus (T2DM), in which insulin resistance might lead to hyperglycemia. There are a total of 383 million people around the world (8.3%) who suffer from T2DM, and it is estimated that the number of patients will reach 592 million by 2035, with a prevalence of 10% [13]. Poor diabetes management is associated with heart disease, stroke, blindness, renal failure, foot amputation, and even death [14]. Diabetes and osteoporosis are both common disease conditions, especially in older patients, and might occur together at times. More than 50 years ago, Albright and Reifenstein proved that diabetes mellitus might possibly show association with bone mass loss, resulting in osteoporosis [14]. Since then, a lot of attention has been paid by several researchers [14–16]. The pathogenesis of low bone density in type 1 diabetes mellitus (T1DM) is related to decreased peak bone mass because of deficiency in insulin and insulin-like growth factors, leading to slow osteoblast growth and poor collagen synthesis [17]. The interaction, in turn, exists between T2DM and bone health due to several factors, including the direct effects of T2DM on bone metabolism and strength and indirect effects of antidiabetic medication-induced altered bone metabolism [18].

However, the research results on the effects of T2DM on bone mineral density (BMD) in clinical epidemiology still remained controversial. Some authors have reported that T2DM is associated with low bone density, few others reported normal bone density, and then few others showed increased BMD [19–21]. Two systematic reviews were conducted in China and Iran, and the pooled prevalence of osteoporosis in T2DM patients in China was 37.8% [22], while the prevalence of lumbar and femoral neck osteoporosis in postmenopausal Iranian women with T2DM was 25.26 and 17.45%, respectively [23]. Although these two reviews claimed that osteoporosis had affected quite a large number of patients with T2DM in China mainland and Iran, these two studies could not still answer the question of whether diabetes mellitus is a risk factor of low bone density?

Hence, this systematic review was conducted to investigate whether diabetes mellitus is a risk factor for low bone density based on a large sample size to provide evidence for physicians as well as the health supervision department.

## Methods

### Evidence acquisition

The guidelines of the Meta-analysis of Observational Studies in Epidemiology Group [24] were followed for the present meta-analysis.

### Data sources and searches

The studies were systematically searched from the PubMed, Embase, CINAHL, and SciELO databases from their inception to January 2020. The search strategy included different combinations of search terms related to risk factors, diabetes, osteoporosis, and low bone density. The search terms used in the present meta-analysis were diabetes mellitus, diabetes, DM, T2DM, type 2 diabetes mellitus, type 2 DM, T1DM, type 1 diabetes mellitus, type 1 DM, osteoporosis, and low bone density in all fields. There was no restriction regarding the country. The reference lists of the retrieved articles and relevant review articles were searched manually for any new studies. In the present meta-analysis, only the data of published articles were included to ensure the quality of studies and results. The search was limited to the English language.

### Inclusion and exclusion criteria

#### Type of studies

Although there are potential limitations for meta-analysis of observational studies, no evidence could be obtained with regard to some areas of health policy from randomized controlled trials. One such example is regarding the association of diabetes mellitus with osteoporosis or low bone density. Thus, cohort studies, cross-sectional studies, and case-control studies were included. If a published study had more than one publication, then the most recent publication or publication with the most complete dataset was selected.

#### Type of participants

Low bone density was defined as BMD values of 2.5 standard deviations below the mean value for young adults (T score ≤ 2.5) based on the lowest T score at the skeletal site according to the International Society for Clinical Densitometry (one diagnostic category) [25]. To ensure the quality of studies and results, the present review included studies with a sample size of over 100 patients.

#### Exposure factors

The factors related to low bone density included diabetes mellitus, irrespective of type 1 diabetes mellitus (T1DM) or T2DM.

#### Outcomes

The outcomes reported were adjusted or non-adjusted odds ratios (ORs) with a corresponding measure of variance or original categorical data related to the risk factors of diabetes mellitus to low bone density.

#### Exclusion criteria

The exclusion criteria were (1) reviews, comments, and lectures, (2) repeated studies, (3) the results in the studies could not be transformed into relative risks (RRs) and their 95% CIs, (4) animal or cell studies, and (5) studies that explored only the mechanism of diabetes mellitus.

### Data extraction

One reviewer (Jingying Qiu) gathered all the papers that presented the risk factors associated with low bone density, including T1DM and T2DM. The studies were selected for inclusion by two reviewers (Jingying Qiu and Zhichun Dong) independently. After deleting the duplications, the titles and abstracts of all identified potential studies were screened. The full texts of all possibly relevant articles were retrieved for comprehensive assessment based on the inclusion criteria. Any disagreements between the two reviewers were resolved by discussion or by reaching a consensus with a third reviewer (Chengjiang Li).

The required data such as the first author, publication year, country, design, sample size, source, sex, and diabetes type, and diagnosis were entered into a pre-designed form. To minimize study selection bias, data extraction and quality evaluation were done by two reviewers (Jingying Qiu and Zhichun Dong) independently. Any disagreements were resolved by consulting a third reviewer (Chengjiang Li).

### Assessment of quality

The quality of included studies was assessed using the Newcastle Ottawa Scale (NOS) for case-controlled studies [24] by two reviewers (Jingying Qiu and Zhichun Dong) independently. Any disagreements were resolved by consulting a third reviewer (Chengjiang Li). NOS for case-controlled studies were assessed based on the following items: (1) 1 point for the adequacy of case definition; (2) 1 point for the representativeness of the cases; (3) 1 point for controls’ selection; (4) 1 point for controls’ definition; (5) 2 points for comparability of the cases and controls according to the design or analysis; (6) 1 point for exposure ascertainment; (7) 1 point for the same method of ascertainment for the cases and controls; and (8) 1 point for the non-response rate) [24]. The studies that scored 5 or more NOS criteria were considered as high quality [26, 27]. As only case-controlled studies were included in this systematic review, only the items of NOS case-controlled studies were listed.

### Statistical analysis

The RevMan analysis software (RevMan 5.3) from the Cochrane Collaboration was used in this meta-analysis. The OR with 95% CI was used to estimate the strength of the association for dichotomous variables. Heterogeneity was quantified using the *I*^2^ index. If the *I*^2^ test indicated significant heterogeneity, i.e., a value > 50%, then a random-effects model was conducted; otherwise, a fixed-effects model was used. Subgroup analysis was conducted based on sex (male or female), economic level (developed or developing countries), and type of diabetes (T1DM or T2DM). A funnel plot was conducted to detect publication bias.

## Results

### Characteristics of studies included

**Fig. 1:**
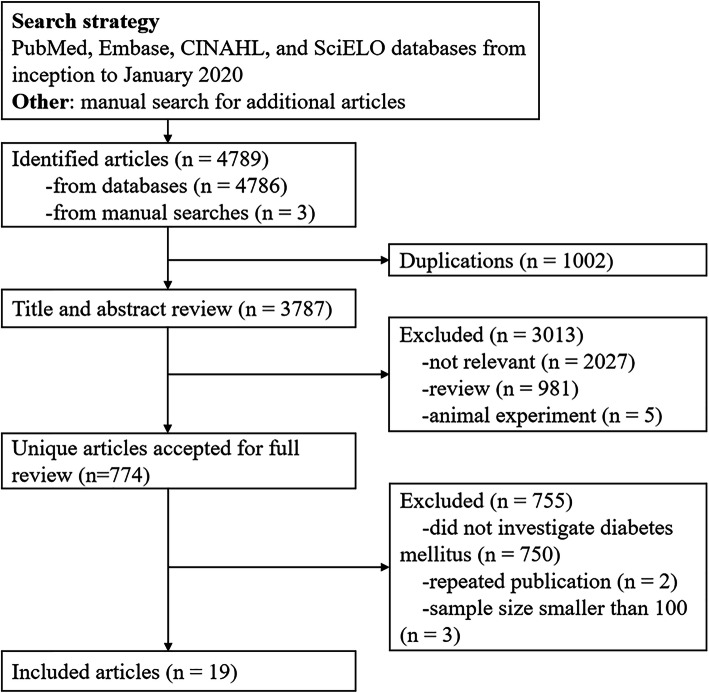
Summary of study identification and selection process

A total of 4789 publications were identified through multiple search engines, and 1002 of these were excluded due to duplications. After examining the titles and abstracts, 3013 articles were excluded. After reviewing the full-texts of the remaining 774 articles, 3 studies were excluded due to sample size smaller than 100, 2 studies were excluded due to repeated publications, 750 studies were excluded as diabetes mellitus was not investigated, and 5 studies were excluded because no information is given about BMD [28–32]. Finally, 14 relevant studies were included in this systematic review [33–46] (Fig. 1).

These 14 case-controlled studies included 24,340 participants, and the sample size ranged from 255 to 6267. Five studies were conducted in China [33, 34, 39, 44, 46], two in Korea [43, 45], two in Jordan [35, 41], and the remaining in other countries. Most of the low bone density patients underwent dual-energy X-ray absorptiometry. Four studies were conducted only in females [35, 38, 40, 41], one study only in males [45], one study reported the data on both females and males separately [33], and the remaining included both females and male participants. Two studies only checked T2DM patients [37, 41], and one study checked T1DM and T2DM separately [40]. The main characteristics of the included studies are presented in Table 1.

**Table 1:** The characteristics of studies included in the meta-analysis

Study	Country	Design	Participant	Diagnose	Sex	Sample size	Diabetes type
Shaw CK 1993 [33]	China-Taiwan	Case-controlled study	Volunteers (15 to 83 years old) living in Lin-Kou Township	BMD of the lumbar spine (L2 to L4) measured by dual-photon absorptiometry (DP4, Lunar Radiation Corp, Madison, WI)	Female and male	404	Both
Chumnumnawin M 2008 [34]	Thailand	Case-controlled study	Priests older than 20 years old to the out-patients Department of Priest Hospital with other complaints	BMD of calcaneum measured by ultrasonography	Both	659	Both
El-Heis MA 2013 [35]	Jordan	Case-controlled study	Women referred to the Radiology Department at King Abdullah University Hospital	BMD of the lumbar spine (L1 to L4) and femoral hip (neck, trochanter) measured by DXA	Female	384	Both
Zhou R 2013 [36]	China	Case-controlled study	Sampled from three randomly selected communities in Chongqing, aged 60 and over	BMD of the femoral neck measured by DXA (Prodigy fan beam densitometer; Lunar, GE Medical System, Madison, WI)	Both	1729	Both
Daisuke A 2015 [37]	Japan	Case-controlled study	Consecutive outpatients aged 50 years at our hospital	BMD of the lumbar spine (L2 to L4) measured by DXA	Both	255	T2DM
Saei GNM 2015 [38]	Iran	Case-controlled study	360 non-pregnant women over the age of 15 who referred for bone density testing to the Urmia Imam Khomeini Academic Hospital	BMD of the femoral neck and the lumbar spine L1-L4 measured by DXA	Female	360	Both
Liu D 2016 [39]	China	Case-controlled study	Local elderly people in 8 communities Chongqing city	BMD of vertebral and femoral neck measured by DXA (GE Medical Systems, Madison, WI)	Both	1802	Both
Neglia C 2016 [40]	Italy	Case-controlled study	Postmenopausal subjects from the Ionian and Salento Osteoporosis Registry/Euro Mediterranean Registry of Osteoporosis	AD-SoS T-score of distal metaphysis of the first phalanges by ultrasound measurements performed by DBM Sonic Bone Profiler 1200	Female	4909	T1/2DM
Dana H 2017 [41]	Jordan	Case-controlled study	All Jordanian women aged over 45 years, of menopausal duration of more than 1 year	BMD of the lumbar spine L1–L4 and left femoral neck measured by DXA	Female	1079	T2DM
Heidari B 2017 [42]	Iran	Case-controlled study	Aged 60 years and older from the Amirkola health and ageing project cohort	BMD of the lumbar spine (L2 to L4) and femoral neck measured by DXA	Both	553	Both
Lee SH 2017 [43]	Korea	Representative case-controlled study	Adults older than 50 years of age from the Korea National Health and Nutrition Examination Survey	BMD of the lumbar spine, femoral neck, and total proximal femur measured by DXA (Hologic Inc., Bedford, MA, USA)	Both	1081	Both
Lin HH 2018 [44]	China-Taiwan	Case-controlled study	Participants older than 50 years, underwent a health examination at a preventive examination agency in urban Taiwan	BMD of the lumbar spine, total hip, and femoral neck measured by DXA (LunarProdigy Advance; GE Healthcare, Madison, WI, USA)	Both	2007	Both
Yoo JE 2018 [45]	Korea	Case-controlled study	Men aged ≥30 years and who underwent dual-energy X-ray absorptiometry in Korean National Health and Nutrition Survey	BMD of the femoral neck, totalfemur, and lumbar spine (L1–L4) measured by DXA (DISCOVERY-W fan-beam densitometer;Hologic, Bedford, MA, USA)	Male	6104	Both
Wang Y 2019 [46]	China	Case-controlled study	General middle-aged and older population in China	Mean BMD measured by DXA (Alara Inc., Fremont, CA, US)	Both	6267	Both

*BMD* bone mineral density, *DXA* dual-energy X-ray absorptiometry

### The quality of the studies

Most of the studies reported the definition of cases. Four studies demonstrated potential selection bias or did not demonstrate the representativeness of the cases [33, 35, 40, 44]. Only one study did not report the details regarding the selection of controls [35]. The comparability was done in all studies. All studies used a structured interview for blinding to case/control status in order to identify the exposure factors. The response rates were reported in all the studies. The overall quality of the studies had a scale of 6 points and was presented in Table 2.

**Table 2:** Quality assessment of studies included in this meta-analysis by Newcastle-Ottawa Scale

Study	Selection				Comparability	Exposure			Quality evaluation
	Cases definition	Cases representativeness	Control selection	Controls definition	Comparability	Ascertainment of exposure	Same ascertainment	Non-response rate	
Shaw CK 1993 [33]	a	b	a	a	b	b	a	a	7
Chumnumnawin M 2008 [34]	a	a	b	a	b	b	a	a	7
El-Heis MA 2013 [35]	a	b	b	a	b	b	a	a	6
Zhou R 2013 [36]	a	a	a	a	a + b	b	a	a	8
Daisuke A 2015 [37]	a	a	b	a	b	b	a	a	7
Saei GNM 2015 [38]	a	a	b	a	b	b	a	a	7
Liu D 2016 [39]	a	a	a	a	a + b	b	a	a	9
Neglia C 2016 [40]	a	b	c	a	b	b	a	a	6
Dana H 2017 [41]	a	a	a	a	b	b	a	a	8
Heidari B 2017 [42]	a	a	a	a	b	b	a	a	8
Lee SH 2017 [43]	a	a	a	a	b	b	a	a	8
Lin HH 2018 [44]	a	b	b	a	b	b	a	a	6
Yoo JE 2018 [45]	a	a	b	a	a + b	b	a	a	8
Wang Y 2019 [46]	a	a	b	a	b	b	a	a	7

### Total

**Fig. 2:**
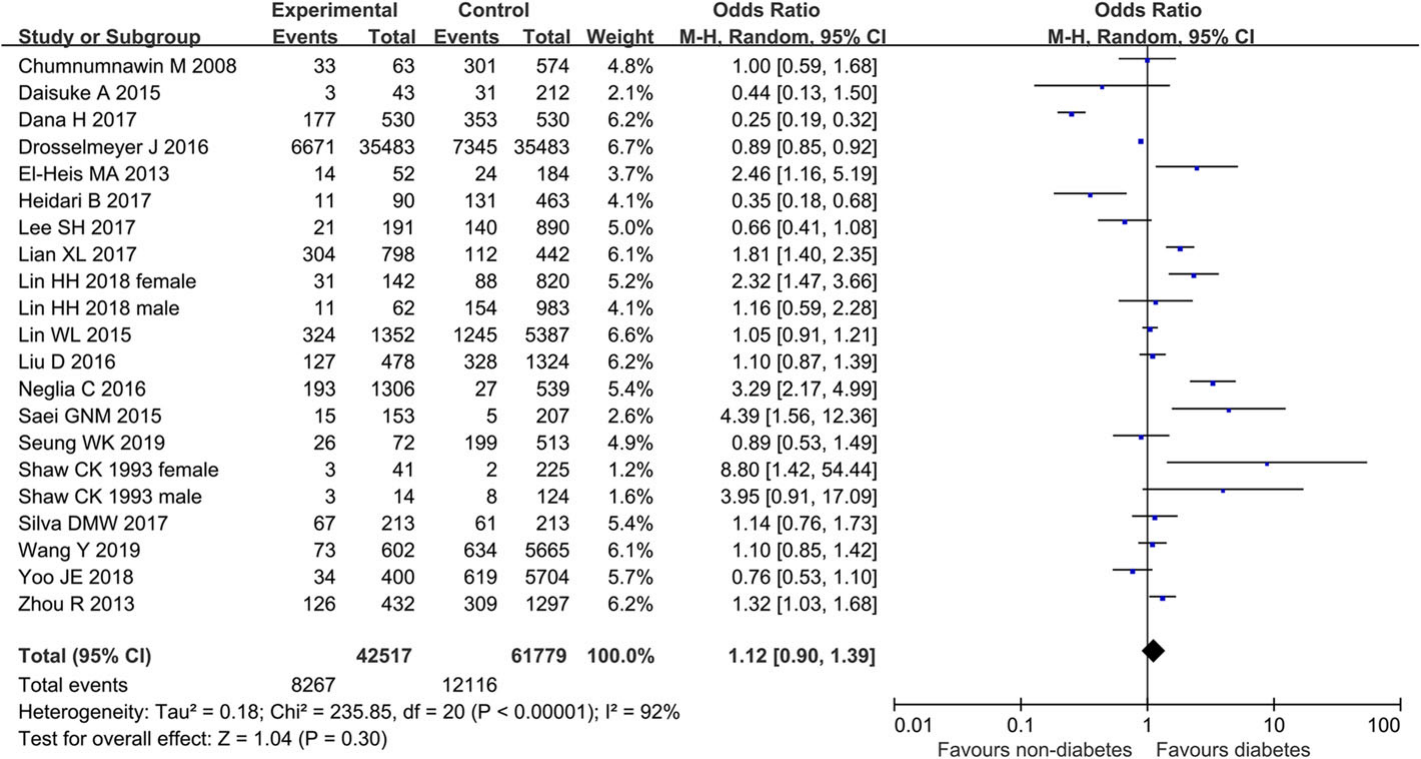
Forest plot on the relationship of diabetes mellitus and osteoporosis

Fourteen studies combined low bone density rates for 4599 diabetic and 19,741 non-diabetic patients. The results showed a statistically significant heterogeneity among the studies (I^2^ = 93%, *P* < 0.00001), and no difference was observed with regard to the prevalence of diabetes between osteoporotic and normal patients (14 studies, *n* = 24,340, OR = 1.20, 95% CI = [0.80, 1.79], *P* = 0.38). Forest plot of diabetes-related to low bone density was presented in Fig. 2.

### Subgroup analysis

Subgroup analysis was conducted based on different populations, economic level, sex, diabetes type, and quality. The results revealed no significant differences regarding the prevalence of diabetes between low bone density and normal patients in Asians (14 studies, *n* = 22,495, OR = 1.10, 95% CI = [0.74, 1.63], *P* = 0.64), but not in Europeans (1 study, *n* = 1845, OR = 3.29, 95% CI = [2.17, 4.99], *P* < 0.00001). The results showed differences with regard to diabetes rate between Asian and European populations (*I*^2^ = 92.8%, *P* = 0.0002).

The studies before 2015 revealed positive correlation between diabetes and low bone density (6 studies, *n* = 3621, OR = 1.76, 95% CI = [1.06, 2.92], *P* = 0.03), while the studies after 2015 showed no significant differences (8 studies, *n* = 20,719, OR = 0.92, 95% CI = [0.53, 1.59], *P* = 0.77).

Four studies were conducted in developed countries, and 10 studies in developing countries. There was no difference regarding the prevalence of diabetes between low bone density and normal patients both in developed (4 studies, *n* = 9285, OR = 0.99, 95% CI = [0.409, 2.41], *P* = 0.97) and developing countries (10 studies, *n* = 15,055, OR = 1.28, 95% CI = [0.80, 2.07], *P* = 0.31). The results showed no significant differences in diabetes rate between developed and developing countries (*I*^2^ = 0%, *P* = 0.61).

Seven studies have reported either in female or male patients only, or separately. There was no difference in the prevalence rate of diabetes between low bone density and normal patients both in females (6 studies, *n* = 4729, OR = 2.21, 95% CI = [0.62, 7.83], *P* = 0.22) and males (3 studies, *n* = 1736, OR = 1.02, 95% CI = [0.31, 3.39], *P* = 0.97). The results showed no differences with regard to diabetes rate between female and male patients (*I*^2^ = 0%, *P* = 0.39).

Three studies reported T1DM or T2DM only, or separately. The results showed differences in the rate of diabetes between low bone density and normal patients both in T1DM (1 study, *n* = 1845, OR = 3.83, 95% CI = [1.64, 8.96], *P* = 0.002), while no difference in T2DM patients (3 studies, *n* = 3160, OR = 0.69, 95% CI = [0.11, 4.55], *P* = 0.70). These results suggested significant differences in the diabetic rate between T1DM and T2DM patients (*I*^2^ = 62.1%, *P* = 0.10).

There was no difference in the rate of diabetes between low bone density and normal patients both in studies with quality of over 7 points (12 studies, *n* = 20,837, OR = 0.94, 95% CI = [0.63, 1.41], *P* = 0.76), and below 7 points, showing significant differences (3 studies, *n* = 4088, OR = 2.27, 95% CI = [1.50, 3.43], *P* = 0.0001). These results suggested significant differences in diabetic rate between studies with quality of over and below 7 points (*I*^2^ = 88.7%, *P* = 0.0001). The results of subgroup analysis were presented in Table 3.

**Table 3:** The results of subgroup analysis

Subgroup	Studies	Heterogeneity analysis	OR	*P* value
Population				
Asian	14	91%, *P* < 0.00001	1.08 [0.74, 1.57]	0.69
European	1	/	3.29 [2.17, 4.99]	*P* < 0.00001
Year				
Before 2015	6	67%, *P* = 0.006	1.76 [1.06, 2.92]	0.03
After 2015	9	95%, *P* < 0.00001	0.92 [0.56, 1.52]	0.75
Sex				
Female	6	97%, *P* < 0.00001	2.21 [0.62, 7.83]	0.22
Male	3	83%, *P* = 0.003	1.02 [0.31, 3.39]	0.97
Economic Level				
Developed countries	5	89%, *P* < 0.00001	0.98 [0.49, 1.96]	0.95
Developing countries	10	93%, *P* < 0.00001	1.28 [0.80, 2.07]	0.31
Diabetes type				
T1DM	1	/, *P* = 0.002	3.83 [1.64, 8.96]	0.002
T2DM	3	98%, *P* < 0.00001	0.69 [0.11, 4.55]	0.70
Quality				
Over 7 points	13	91%, *P* < 0.00001	0.89 [0.67, 1.17]	0.40
Below 7 points	6	86%, *P* < 0.00001	1.71 [1.19, 2.45]	0.004

*OR* odds ratio, *T2DM* type 2 diabetes mellitus, *T1DM* type 1 diabetes mellitus

### Sensitivity analysis

Sensitivity analysis revealed that after excluding 8 studies [26, 32, 33, 35–37, 39, 41], the results still remained the same (11 studies, *n* = 25,861, OR = 1.05, 95% CI = [0.91, 1.20], *P* = 0.52).

### Publication Bias

**Fig. 3:**
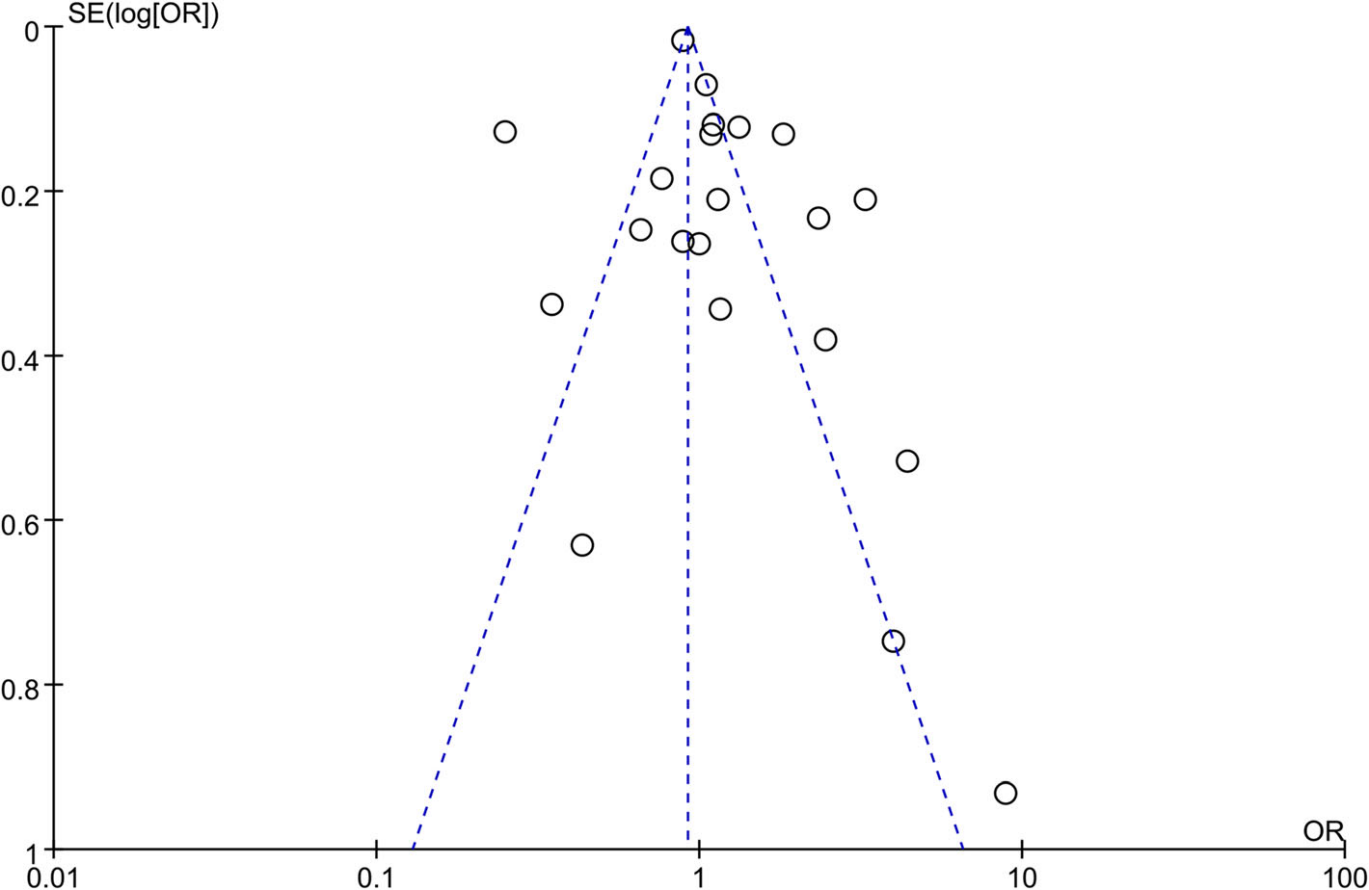
Funnel plot for publication bias

No significant asymmetry was observed in the funnel plot (Fig. 3).

## Discussion

This is the first report based on the epidemiological data to study whether diabetes mellitus is a risk factor for low bone density. A total of 14 articles with 24,340 participants were included in this review, and the results suggested that the evidence regarding the relationship between diabetes mellitus and low bone density still remains to be inadequate. The overall quality of each study was over 6 points, and so publication bias was ruled out. Subgroup analysis revealed that females or males, developed or developing countries and T2DM patients showed no significant difference with regard to the risk of low bone density, and also, the evidence between T1DM and low bone density remains to be inadequate. The damage of T1DM and T2DM on bone health was different.

### Low bone density with type 1 diabetes mellitus

T1DM, which is also considered insulin-dependent diabetes, occurs when insulin is insufficient and causes hyperglycemia in young patients [47, 48]. It is usually diagnosed in childhood or early adulthood. Only one study explored the risk factor of T1DM to low bone density, and so this systematic review could not provide evidence of whether T1DM acts as a risk factor for osteoporosis. Previous studies have shown that the risk of hip fracture is increased in both T1DM and T2DM patients, whereas low bone density based on bone scans occurs more frequently in T1DM than expected, while the incidence in T2DM patients is lower [49]. The latter might be due to the differences in body size, i.e., the T2DM patients were more obese or had a higher body mass index than the general population, and BMD increases with increasing body size [50]. T1DM occurs due to overall poor bone health and lower peak bone mass in adolescents and is mainly manifested due to the following aspects: (1) abnormal growth hormone (GH)-insulin-like growth factor-1 (IGF-1) axis, which leads to bone loss before the bone reaches to its peak mass in a decreased adolescent growth potential [51, 52]; (2) disobeying planned medical management, making metabolic disorder worse [53, 54]; (3) dietary control leads to insufficient dietary calcium intake [55]; and (4) increased urinary calcium excretion [56]. In addition, individuals with T1DM were associated with an increased risk of celiac disease, leading to intestinal malabsorption, poor growth, and low bone density [57]. While the insulin to control glucose in T1DM patients was reported to improve bone health [21].

### Low bone density with type 2 diabetes mellitus

Several studies indicated that despite good BMD results, T2DM patients showed an exact association with a higher risk of fragility fractures. However, some other studies have reported that diabetes mellitus patients are associated with fractures due to lower BMD than those who did not. In this study, only three studies have reported the risk factors of T2DM to low bone density, showing a negative association. In addition, there are bone structural abnormalities, such as obvious loss of trabecular bone loss but decreased cortical BMD and increased cortical porosity, which in turn decreases the bone strength that is associated with lower strength stress index [58]. Lower strength stress index is an important predictor of fracture [59, 60]. Also, the pathogenesis might vary in different T2DM patient populations, which might be due to obesity, old age, diabetic complications, duration, and medication. T2DM is a variable disease condition, and similar is the condition with osteoporosis. The best example is the effect of peroxisome proliferator-activated receptor-γ (PPAR-γ) agents such as thiazolidinediones (TZDs), rosiglitazone, and pioglitazone [61]. These agents might not be so commonly used at the moment now as the former has been removed from the market, and the latter was reduced in application due to side effects, such as increased fracture risk. In bone, PPAR-γ controls the differentiation of mesenchymal and hematopoietic cells. The activation of PPAR-γ by TZDs causes an imbalance in bone remodeling, increases bone resorption, and decreases bone formation. Laboratory studies suggested that the use of selective PPAR-γ modulators can separate the harmful effects of PPAR-γ on the bone from its beneficial antidiabetic effects [61]. It was found that women with T2DM who initiated insulin intake experienced more rapid BMD loss at the femoral neck when compared to those women who did not use insulin [62].

### Limitation of this review

This meta-analysis is conducted based on several case-controlled studies in many different countries, and included a relatively large sample size, and ruled out publication bias. However, the evidence collected is still limited. Firstly, the case-controlled studies were less expensive and time-consuming and required the collection of a larger mass of data with a low evidence level. Also, most of the studies were single-center studies, and so the conclusions still need further confirmation. In addition, due to the different characteristics of the studies, heterogeneity was considered inevitable, although sensitivity analysis could not change the results. The potential sources of heterogeneity mainly occur due to different types of diabetes mellitus or other reasons. What’s more, the included studies evaluated BMD at different bone sites with different techniques (DXA and ultrasonography or radiographic absorptiometry) and so are not adequately comparable.

## Conclusions

This systematic review and meta-analysis confirmed that the evidence regarding the relationship between diabetes mellitus and low bone density is inadequate. Subgroup analysis revealed that whether females or males, developed or developing countries and T2DM patients showed no significant differences with regard to the risk of low bone density, and also between T1DM and low bone density. Due to differences in T1DM and T2DM, and due to close relation to female menopause, our study suggested that the reporting of the details separately in females and males, and it should be clear whether it is T1DM or T2DM. So, well-designed cohort studies are expected in the future for further confirmation of our study results.

## Data Availability

The datasets used and/or analyzed during the current study are available from the corresponding author on reasonable request.

## Abbreviations

NOS: Newcastle-Ottawa Scale
OR: Odds ratio
CI: Confidence interval
PTH: Parathyroid hormone
T2DM: Type 2 diabetes mellitus
BMD: Bone mineral density
DXA: Dual-energy X-ray absorptiometry
TZDs: Thiazolidinediones
